# Skeletal fluorosis from the point of view of an occupational exposure to fluorides in former Czechoslovakia

**DOI:** 10.2478/v10102-010-0038-7

**Published:** 2010-11

**Authors:** Jana Buchancová, Hubert Poláček, Henrieta Hudečková, Lukáš Murajda, Oto Osina, Jela Valachová

**Affiliations:** 1 Institute of Public Health, Jessenius Faculty of Medicine Comenius University, Martin, SLOVAKIA; 2 Radiodiagnostics Clinic, Jessenius Faculty of Medicine Comenius University, Martin, SLOVAKIA; 3 Clinic of Occupational Medicine and Toxicology, Jessenius Faculty of Medicine Comenius University, Martin, SLOVAKIA

**Keywords:** health status, fluorides in urine, hypocalcaemia, x-ray evaluation of skeleton

## Abstract

Electrolytic production of aluminium in former Czechoslovakia started in the year 1953 in the Žiar valley in the central Slovakia. However, till 1995 the hygienic conditions for health protection were not met in the factory. It underwent a reconstruction afterwards.

The authors demonstrate cases of occupational skeletal fluorosis (currently rare in Europe) in 14 metallurgists which were all disclosed in foundry workers in Žiar nad Hronom as to the year 2005. The occupational disease was diagnosed after 17.7 ± 7.67 years (x±SD) of exposure in the foundry.

The authors describe the clinical conditions, haematological and biochemical tests (decreased level of ionising calcium was found in serum). The content of fluorides in urine was increased (254.4±130.95 µmol/l). The average age of patients at the time of recognition of the professional etiology of the disease was 57.93±7.95 years. Eight patients were older than 60 years. Skeletal abnormalities were evaluated by using X-ray skiagraphy, estimating the Stage I–III of the skeletal fluorosis. Typically an increase of bone density was found, the compact part of long bones was coarsed, there were calcifications of the interosseous membrane between radius and ulna and some ossifications of the sacrospinal and sacrotuberous ligaments. Twelve patients suffered sensorimotor polyneuropathy of extremities, chronic bronchitis was found in 6 patients (two of them were smokers).

The last occupational case was registered in the year 2001. The authors assume that aluminium production with modern technology of better safety and protection of health of workers is the key which will make the skeletal fluorosis the history in the Czech and Slovak Republic.

## Introduction

The accumulation of fluorides in the organism is usually a long-term process. It appears only after many years of exposure as a bone-joint impairment, with characteristic x-ray signs of bone fluorosis and possibly as a secondary impairment of the nervous system (Liteplo *et al*., 2002). Existing bone hyperostosis can sometime narrow the spaces around peripheral nerves. The storage of fluorides may increase for example in the aorta and other organs.

Aim of the work was to perform a detailed retrospective analysis of health status of a group of 14 foundry workers who were diagnosed for professional bone fluorosis during a 50-year period of existence of a factory with electrolytic aluminium production in former Czechoslovakia and to point out this occurrence which is exclusive in past decades in Europe.

## Professional exposition to fluorides in foundry industry

Professional exposition to fluorides was first registered by Moller and Gudjonsson in 1932 in foundry workers at fluorspar (CaF_2_) processing in steel industry. Skeletal fluorosis was present in one fourth of workers in aluminium metallurgy in the past (Knight, 1988). At the aluminium electrolytic production the aerosols with content of fluoride or hydrogen fluoride get to the indoor air of production halls from cryolite Na_3_AlF_6_ and AlF_3._ Fluorides of natrium and calcium are added to melting to meliorate the solubility of cryolite and decrease the melting point of the electrolyte.

## Working conditions in the factory

The factory for electrolytic production of aluminium in the Žiar valley was established in 1953. From the beginning the conditions for health protection were unsatisfactory, the efficiency of suction was insufficient; there was a lack of finances for reconstruction of a bad technology. The average values of fluorides in production halls‘ air were measured irregularly. E.g. in 1960–68 it was 3.69mg/m^3^ of air, in 1987–89 between 0.16–13.7mg/m^3^ of air. Values of aluminium were not measured. The working week in foundry lasted for 36 hours, 6 hours per day, with one free day in the week. The professions in the foundry were: smelter, crane operator, welder, feeder of raw materials, puncher, caster, metal drawer and fireclay-bricklayer. Further details about risk factors of the working environment and about health of the workers were not open to public till 1990. In 1970 was published awork of Lányi and Geryk on the skiagraphy of the spinal column and pelvis in 400 foundry workers of the factory. The increase of x-ray changes of the skeleton was linked to age. In 1985 Vido *et al*. cautiously gave notice in journal *Pracovní lékařství* about possible x-ray signs of bone fluorosis. There was a large reconstruction of the aluminium foundry only in 1995. The production of aluminium has a completely new technology with closed electrolysers since then. Compared to the past when there were 20.2 kg of fluorides released per ton of aluminium produced, at present there is an amount of 0.32 kg of fluorides released per 1 ton of aluminium. In 2004 Slovakia with this factory with 160,000 tons of Al/yr was at the 12th place in Europe in production of aluminium (Černaj, 2005).

## Material and methods

We included 14 patients in the group who were received at the Clinic of occupational medicine and toxicology JFM CU in Martin with the diagnosis of professional bone fluorosis. They were all the cases of since the establishment of the factory. First 6 cases were registered in 1965–69; next 8 cases appeared gradually until 2005. There was an x-ray skiagraphy of thorax, the diaphyses of humerus, both forearms, the diaphyses of legs, the pelvic bones and lumbosacral part of the axial skelet performed in all the workers. ECG, spirometry, orthopedic and neurological examination was performed, too. Some selected hematological and biochemical parameters were assessed (BSR, blood count, glycaemia fasting, creatinine in serum, transaminases, uric acid, latex test, total and ionized calcium, phosphorus in serum and alkaline phosphatase in serum).

Fluorides in urine were measured electrotermically from a 10 ml urine sample. In our area the mean values of fluorides in urine range up to 50µmol/l of the urine. An indicative biological cut-off value at sampling aftershift was 560µmol/l of the urine, before next shift 320µmol/l of the urine (Kernová, 2003).

X-ray manifestation of bone fluorosis was evaluated by using criteria according to Hagen and Grinsberg (1970).

Stage I: Bone thinning, ill-defined margins, gradually changed for increased density of bone tissue; Stage II: coarsening of the corticalis of spongiuos bones and periostal appositions; Stage III: marked density, loss of trabecular structure, periostoses and osteophytes formation, ossification and calcification of the ligaments, typically in sacroiliacal region (approx. after 15 years of work in risk).

As typical manifestation we may consider the calcifications of interosseal membrane between the radius and ulna, calcifications of fascia between the fibula and tibia, sometimes curtains of apositions coming out from lower parts of ribs, calcificated intervertebral ligaments, formation of pathological fractures.

## Results

Mean age of the group of men at registration of professional diseases was 57.9±7.9 years (x ±SD), mean exposure in aluminium foundry was 17.7±7.7 years (x±SD).

Half of the patients reported pain, stiffness, reduction of movement of the lumbosacral spine, difficulties at walking, burning pain in frontal part of the lower leg, pain of forearm muscles, extremities‘ muscles cramps in connection to augmented sweating with documented hypocalcaemia. Pain of joints of the extremities was only in 3 men of the sample. Fatigue was reported by ahalf of group. Six workers suffered from chronic bronchitis. Twelve patients were affected by moderate to mid sensomotoric polyneuropathy. All the followed had the BMI over 26.9, three had diabetes mellitus type 2 and one had an impaired glucose tolerance. The latex values were negative among all, creatinine, uric acid in serum and transaminases were normal. Selected laboratory results are shown in Table 1.

**Table 1:** Selected laboratory findings (x ± SD) among the group of patients with the diagnosis of bone fluorosis.

Parameter	Values			

	X ± SD	Min.	Max.	Normal Range
Hemoglobin(g/l)	138.59±26.75	84.00	161.00	130–160
Glycaemia fasting(mmol/l)	5.71±1.63	3.92	7.92	3.3–5.5
Serum cholesterol(mmol/l)	6.30±1.25	3.80	7.92	2.8–5.2
Serum calcium(mmol/l)	2.4±0.14	2.20	2.65	2.15–2.55
Ionized serum calcium(mmol/l)	1.07±0.07	0.95	1.14	2.15–2.55
Serum phosphorus(mmol/l)	0.94±0.16	0.71	1.16	0.87–1.45
Alkaline phosphatase(kat/l)	1.04±0.44	0.50	1.46	0.8–2.2
Fluorides in urine(mol/l)	254.54±130.95	32.00	491.10	up to 50

(n=14)

Mean values of calcium and phosphor in serum were at lower border of the referential interval, the values of ionized calcium were low.

**Figure 1:**
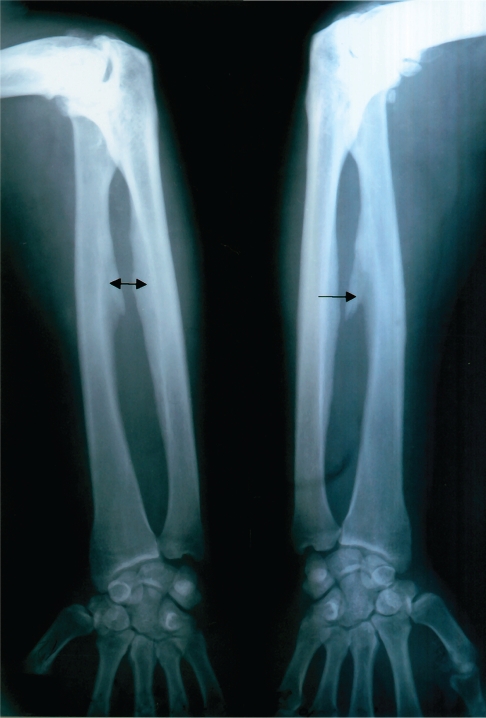
Skeletal fluorosis (Stage II–III). Osteosclerosis of diaphyses of forearm and calcification in membrana interossea (arrows).

**Figure 2:**
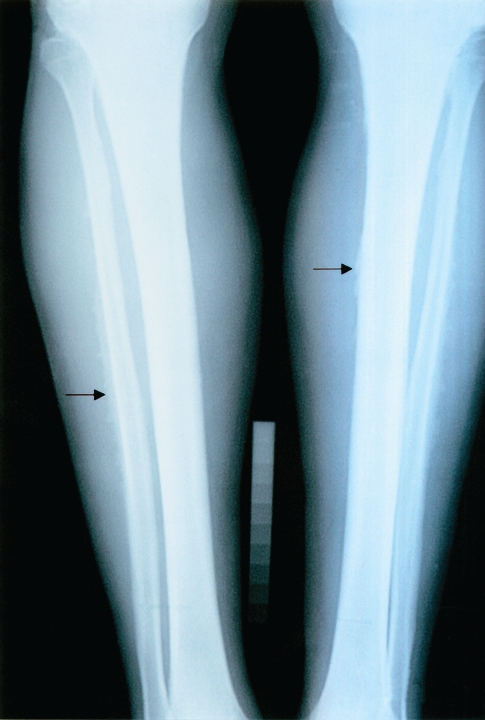
Skeletal fluorosis (Stage II). Tibiae and fibulae thinning, ill defined margines (arrows).

**Figure 3:**
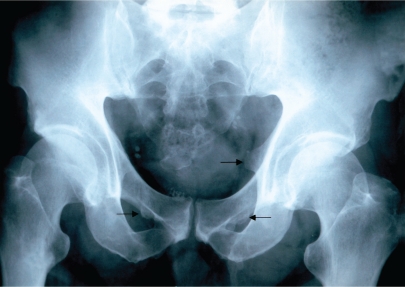
Skeletal fluorosis (Stage III). Exostoses in area of foramen obturatorium dx. I (low arrows).

**Figure 4:**
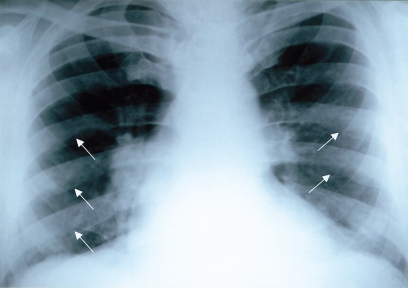
Skeletal fluorosis (Stage III). Pseudoperiostoses of lower margines of ribs (arrows).

Mean values of fluorides in urine give evidence of a 5-time exceeding of the values in non-exposed population. In 3 cases the indicative biological cut-off value was exceeded. Bone fluorosis in the group was assessed after occupational-medical examination, objectivisation of the exposure, according to x-ray findings by radiological specialists as: Stage I in 9 cases, Stage II in 3 cases, Stage II–III in 2 cases. Typical x-ray findings were the calcifications in the area of interosseal membrane between radius and ulna (Figure 1) and between tibia and fibula, tendinous exostoses in the area of foramen obturatorium of the pelvic bones (Figure 3), ossifications in area of spina iliaca anterior et superior, coarsening of compacta of the long bones of limbs with narrowing of the bone marrow cavity (Figure 2) and pseudoperiostoses of lower margines of ribs (Figure 4). In some cases it was augmentation of a density of the skelet in lumbosacral and pelvic areas and different osteophytic periostoses. Four patients had dense flebolites on x-ray of the pelvic area.

Eight patients were retired, six workers (48–53-year-old) with abone fluorosis Stage I continued working after registration of the occupational disease, already in better working conditions. Health status of the whole group was continuously followed-up via Clinic of Occupational Medicine and Toxicology JFM CU in Martin approximately the 5–10 years. In 8 patients the x-ray was performed only in selected localisations of the skelet in 2–4-year periods. In 2 cases amild drop of the bone density in area of the spine was found after two years. In other patients the x-ray finding stayed unchanged in the examined segments of the skelet. After two years from the break of the exposure the values of fluorides in urine were constantly under the indicative cut-off limit.

## Discussion

A retrospective assessment of cases of professional fluorosis is to some extent influenced by the fact, that it is not possible to evaluate the disease diagnosed some decades ago using avariety of methods as it is at present when there is an opportunity to perform for example densitometry, HRCT and other. Therefore the authors introduced the basic laboratory screening and assessed the skiagrams of typical areas of skeleton which brought information about different seriousness of manifestation of the stages of fluorosis. The importance of sensitivity of x-ray diagnostic equipment at suspection for fluorosis was underlined as well by Czervinski *et al*. (1988). In the past 15 years when the professional exposure to fluorides is lower, there has been atendency to avoid the x-ray examination of the skelet from preventive reasons and to perform it by selection – in aluminium foundry workers not until 10–15 years from the start of exposure to fluorides. The evaluation of working environment, preventive medical examination of the workers with follow-up of the content of the fluorides in urine, preferably aftershift, stay obligatory and the content of fluorides is counted to the amount of creatinine in urine. The content of aluminium is estimated by the AAS method (Valachová, 2006).

In the group of our patients there was found one fracture of the collum femori. Although it was at a fluorosis Stage I, it couldn't be directly linked to the exposure to fluorides. A moderate sensomotoric impairment of peripheral nerves was afrequently observed manifestation. It was mainly on upper extremities, what can be related to excessive physical overloading of the extremities, in 2 cases also to the work with vibration tools. Some role could be played by the microclimate in the foundry, in some cases ametabolic syndrome, an influence of diabetes mellitus.

There is evidence in the literature that aconsiderable portion of the fluorides can be eliminated from body by intense sweating (even 1/3 of daily intake – Knight, 1988).

Three of our patients reported marked sweating, their values of fluorides in urine were not lower than in other members of the group, though. All the patients had considerably higher BMI. The fluorides storage in skeleton and the binding with calcium increases the weight of the skeleton. However, it could not be verified if this value was transmitted to the BMI. Regarding the higher values of cholesterol the higher BMI can be considered as an obesity marker.

According the x-ray specialists the calcificates in the minor pelvis are not rare also without the exposure to fluoride. They are often flebolites. Three of our patients had in their personal history attacks of urolithiasis. Repeated higher content of fluorides in urine supported the diagnosis. If the actual value of fluorides was not increased, in retired person with typical x-ray finding and considerable and long-term exposure in the aluminium foundry the final diagnosis was not excluded. These circumstances were stressed by other authors, too (Liteplo *et al*., 2002). The age factor and demineralising osteoporotic tendency could influence the lower values of ionized calcium.

Some added environmental exposure to fluorides may rise from the coal combustion. In territories where this is common, it can be the main source of fluorine pollution (*http://www.floridealert.org/fluorosis-india.html*). The coal contains on average 0.01% of fluorine. Commonly used coal in Slovakia has 20–40% ash particles which contain approximately 400–560mg of fluorides/kg, most often 0.05% of fluorine (Turčanová, 1994). The data about fluorine content in the outdoor air or the drinking water of our patients were not known. The importance of added non-professional exposure from some sources (mineral water, tea) may not be minor at ad hoc evaluation of the fluorine content in urine (Kotěšovic *et al*., 1987). In the environment of the Žiar valley, in the surrounding of the aluminium foundry there were no cases of fluorosis registered in the population. Manifestations of aluminiosis did not occur in the factory.

Professional bone fluorosis is becoming more clinically important usually at the age over 60 years. In our follow-up of the foundry workers it was not connected with a significant reduction of movability nor with significant joint pain. This is expressed in low evaluation of financial compensations in case of an occupational disease registration.

Since an effective treatment is not known, new technologies of production of aluminium which provide an incomparably better health protection are welcome.

## References

[CIT0001] Černaj I (2005). Ekologická výroba hliníka pomohla celej Žiarskej kotline. Trend.

[CIT0002] Czerwinski E, Nowack J, Dabrowska SD, Skolarczyk A, Kita B, Ksiezik M (1988). Bone and joint pathology in fluoride–exposed workers. Arch Environ Health.

[CIT0003] Hagen I, Ginsberg AV, Lányi A. cit., Geryk B, Kernová M, Buchancová J, Klimentová G, Šulcová M, Fabianová E (2003). Biological exposure tests in occupational medicine and in toxicology. Occupational medicine and toxicology.

[CIT0004] Kernová M, Buchancová J, Klimentová G, Šulcová M, Fabianová E (2003). Biological exposure tests in occupational medicine and in toxicology. Occupational medicine and toxicology.

[CIT0005] Knight AI, Zenz C (1988). Fluorides. Occupational Medicine II.

[CIT0006] Kotěšovic F, Míšková I, Brynda J (1987). Evaluation of unoccupational loading of fluorides – necessary assumption for evaluation of occupational exposure – 1st part. Pracov Lék.

[CIT0007] Lányi A, Geryk B (1970). Verknöchterungen der Ansatzstellen einiger Sehnen und Bänder und ihr differentialdiagnostischer. Wert Rad Diagn.

[CIT0008] Liteplo R, Howe P, Malcolm H, international group of experts (2002). Fluorides. Environmental Health criteria.

[CIT0009] Moller PF, Gudjonsson SV (1932). Massive fluorosis of bones and ligaments. Acta Radiol.

[CIT0010] Valachová J (2006). Toxicological profile of aluminium. PhD postgraduate work JLF UK Martin, 2006.

[CIT0011] Vido A, Vido M, Nárožná Z (1985). Röntgenological findings in movement apparatus in foundry workers in alluminium production after 20 years of exposure. Pracov Lék.

